# Truncation of type IV pilin induces mucoidy in *Pseudomonas aeruginosa* strain PAO579

**DOI:** 10.1002/mbo3.86

**Published:** 2013-03-27

**Authors:** T Ryan Withers, F Heath Damron, Yeshi Yin, Hongwei D Yu

**Affiliations:** 1 Department of Biochemistry and Microbiology, Joan C. Edwards School of Medicine at Marshall UniversityHuntington, West Virginia, 25755-9320; 2 Department of Pediatrics, Joan C. Edwards School of Medicine at Marshall UniversityHuntington, West Virginia, 25755-9320; 3 Progenesis Technologies, LLC1111 Veterans Memorial Blvd, Huntington, West Virginia, 25701

**Keywords:** Alginate, biofilms, *muc-23*, *pilA*, *Pseudomonas aeruginosa*

## Abstract

*Pseudomonas aeruginosa* is a Gram negative, opportunistic pathogen that uses the overproduction of alginate, a surface polysaccharide, to form biofilms in vivo. Overproduction of alginate, also known as mucoidy, affords the bacterium protection from the host's defenses and facilitates the establishment of chronic lung infections in individuals with cystic fibrosis. Expression of the alginate biosynthetic operon is primarily controlled by the alternative sigma factor AlgU (AlgT/σ^22^). In a nonmucoid strain, AlgU is sequestered by the transmembrane antisigma factor MucA to the cytoplasmic membrane. AlgU can be released from MucA via regulated intramembrane proteolysis by proteases AlgW and MucP causing the conversion to mucoidy. *Pseudomonas aeruginosa* strain PAO579, a derivative of the nonmucoid strain PAO1, is mucoid due to an unidentified mutation (*muc-23*). Using whole genome sequencing, we identified 16 nonsynonymous and 15 synonymous single nucleotide polymorphisms (SNP). We then identified three tandem single point mutations in the *pilA* gene (PA4525), as the cause of mucoidy in PAO579. These tandem mutations generate a premature stop codon resulting in a truncated version of PilA (PilA^108^), with a C-terminal motif of phenylalanine-threonine-phenylalanine (FTF). Inactivation of *pilA*^108^ confirmed it was required for mucoidy. Additionally, *algW* and *algU* were also required for mucoidy of PAO579. Western blot analysis indicated that MucA was less stable in PAO579 than nonmucoid PAO1 or PAO381. The mucoid phenotype and high P_*algU*_ and P_*algD*_ promoter activities of PAO579 require *pilA*^108^, *algW*, *algU*, and *rpoN* encoding the alternative sigma factor σ^54^. We also observed that RpoN regulates expression of *algW* and *pilA* in PAO579. Together, these results suggest that truncation in type IV pilin in *P. aeruginosa* strain PAO579 can induce mucoidy through an AlgW/AlgU-dependent pathway.

## Introduction

Cystic fibrosis (CF) is a genetic disorder that results from mutations in the CF transmembrane conductance regulator gene (Rommens et al. 1989). These mutations cause a disruption in chloride transport of mucosal tissues resulting in an accumulation of dehydrated mucus. This accumulation of mucus within the lungs prevents the removal of infectious agents by interfering with the mucocilliary escalator (Chmiel and Davis 2003). This provides a hospitable environment for the adherence and cultivation of microbial pathogens (Bauernfeind et al. 1987; Saiman et al. 1992). As a result, individuals afflicted with CF are highly susceptible to various bacterial infections including *Pseudomonas aeruginosa* (Govan and Deretic 1996). *Pseudomonas aeruginosa* is a Gram negative, opportunistic pathogen that uses the overproduction of alginate, a surface polysaccharide, to form biofilms. The overproduction of alginate, also known as mucoidy, is responsible for the establishment of chronic infections, as well as an increased resistance to antibiotics (Govan and Deretic 1996) and phagocytosis by macrophages (Leid et al. 2005) in CF patients. Chronic lung infections with *P. aeruginosa* cause an increase in morbidity and mortality in individuals afflicted with CF (Lyczak et al. 2002), and this transition from the nonmucoid to the mucoid phenotype is a proven predictor of an overall decline in the patient's health (Henry et al. 1992).

Typically, constitutively mucoid strains arise in the lungs of CF patients due to mutations in the *mucA* gene, which encodes the inner membrane-spanning antisigma factor (Martin et al. 1993; Boucher et al. 1997). MucA is a negative regulator of alginate overproduction because it sequesters AlgU (AlgT, σ^E^, σ^22^), the primary sigma factor responsible for activation of the alginate biosynthetic operon at the *algD* promoter (Wozniak and Ohman 1994). Alternatively, the conversion to mucoidy can occur when MucA is degraded by regulated intramembrane proteolysis (Qiu et al. 2007). Proteolytic degradation is initiated through cleavage of the C-terminal of MucA between the alanine and glycine residues at position 136 by the serine protease AlgW (Cezairliyan and Sauer 2009), anchored in the periplasmic leaflet of the inner membrane, and followed by the transmembrane protease MucP (YaeL) and the cytoplasmic proteases ClpX and ClpP (Qiu et al. 2007, 2008b; Cezairliyan and Sauer 2009). The activation of AlgW, and subsequent proteolysis of MucA, is thought to be in response to extracellular stress, as well as the accumulation of misfolded envelope proteins (Qiu et al. 2007; Wood and Ohman 2009). We previously found that induction of a small envelope protein called MucE causes mucoidy (Qiu et al. 2007). MucE has an AlgW activation signal with a C-terminal motif of tryptophan-valine-phenyalanine (WVF) (Qiu et al. 2007). The MucE peptide has also been shown to be a potent ligand to activate AlgW to degrade the periplasmic fragment of MucA (Cezairliyan and Sauer 2009).

*Pseudomonas aeruginosa* strain PAO579 was first generated in the 1970s through the isolation of mucoid variants of PAO381 (Govan and Fyfe 1978), a nonmucoid derivative of the progenitor strain PAO1, following exposure to carbenicillin. PAO579 is highly mucoid due to unclassified mutation(s) that is referred to as *muc-23* (Govan and Fyfe 1978). Previously, it has been shown that mucoidy in PAO579 depends on the alternative sigma factor RpoN (σ^54^) (Boucher et al. 2000). In this study, we used whole genome sequencing to identify mutation(s) that cause the mucoidy of PAO579. We identify three tandem mutations in *pilA* that are responsible for the mucoid phenotype in this strain. Moreover, the mucoid phenotype of strain PAO579 is dependent upon AlgW, as well as AlgU and RpoN. Our data suggests truncation of pilin induces mucoidy in *P. aeruginosa* strain PAO579.

## Experimental Procedures

### Sequence analysis of PAO579

Methods and parameters used in the sequencing of *P. aeruginosa* strain PAO579 were previously described (Withers et al. 2012). The *pilA*^108^ gene of PAO579, which was also sequenced by the Marshall University Genomics Core Facility, has been separately deposited to GenBank under the accession number KC692835.

### Bacterial strains and growth conditions

Bacterial strains used in this study are indicated in Table 1. *Pseudomonas aeruginosa* and *Escherichia coli* strains were grown at 37°C in Lennox broth (LB), on LB agar or *Pseudomonas* Isolation Agar (PIA). When indicated, the media was supplemented with carbenicillin, gentamycin, tetracycline, kanamycin, and/or arabinose.

**Table 1:** Bacterial strains and plasmids used in this study

Strain, plasmid	Genotype, phenotype, description	Reference
*Escherichia coli*		
TOP10	DH5α derivative	Invitrogen
*Pseudomonas aeruginosa*		
PAO1	*algU*^+^*mucA*^+^; nonmucoid	P. Phibbs*
PAO381	*algU*^+^*mucA*^+^; nonmucoid, derived from PAO1	J. Govan**
PAO579	*algU*^+^*mucA*^+^*muc-23*; mucoid, derived from PAO381	J. Govan**
PAO579Δ*algU*	*mucA*^+^*muc-23*, In-frame deletion of *algU* (PA0762); nonmucoid	This study
PAO579Δ*algW*	*algU*^+^*mucA*^+^*muc-23*, In-frame deletion of *algW* (PA4446); nonmucoid	This study
PAO579*pilA*::*aacC1*	*algU*^+^*mucA*^+^*muc-23*, *pilA*::Gm^R^ (PA4525) encoding a type IVa pilin precursor; nonmucoid	This study
PAO579*rpoN*::Tc^R^	*algU*^+^*mucA*^+^*muc-23*, *rpoN*:: Tc^R^ (PA4462) of the sigma factor RpoN (σ^54^); nonmucoid	This study
Plasmids		
pCR4-TOPO	3.9-kb, Ap^R^, Km^R^; TA cloning vector	Invitrogen
pRK2013	Km^R^, *Tra Mob ColE1*	(Figurski and Helinski 1979)
pHERD20T	pUCP20T P_*lac*_ replaced by 1.3-kb AflII-EcoRI fragment of *araC*-P_*BAD*_ cassette	(Qiu et al. 2008a)
pHERD20T-*algW*	*algW* (PA4446) from PAO1 in pHERD20T; EcoRI/HindIII	This study
pHERD20T-*algW* ^I239F^	*algW* (PA4446) from PAO579 in pHERD20T; EcoRI/HindIII	This study
pHERD20T-*pilA*	*pilA* (PA4525) from PAO1 in pHERD20T; EcoRI/HindIII	This study
pHERD20T-*pilA*^108^	*pilA* (PA4525) from PAO579 in pHERD20T; EcoRI/HindIII	This study
pHERD20T-*pilA*-HA	C-terminally tagged *pilA-*HA ending with the PKGCDN motif cloned in pHERD20T; EcoR1/HindIII	This study
pHERD20T-*pilA*^108^-HA	C-terminally tagged *pilA*-HA ending with the DITFTF motif cloned in pHERD20T; EcoR1/HindIII	This study
pHERD20T-*oprF*	*oprF* (PA1777) from PAO1 in pHERD20T; EcoRI/HindIII	This study
pHERD20T-*oprF*-FTF	*oprF* (PA1777) from PAO1 with FTF-motif fused to the C-terminal; EcoRI/HindIII	This study
pHERD20T-HA-*mucA*	N-terminally tagged HA-*mucA* in pHERD20T; EcoRI/HindIII	(Damron et al. 2009)
pUCP20T-P_*BAD*_−*rpoN*	*araC*-P_*BAD*_-*rpoN* fusion in pUCP20; XbaI/HindIII	(Damron et al. 2009)
miniCTX-*lacZ*	Gene delivery system used to fuse target genes to *lacZ* and integrate onto the chromosome at the CTX phage *att* site in *Pseudomonas aeruginosa*, Tc^R^	(Hoang et al. 2000)
miniCTX-P_*algD*_−*lacZ*	Complete P_*algD*_ promoter (1525 bp upstream of ATG) HindIII/BamHI in miniCTX-*lacZ*	(Damron et al. 2009)
miniCTX-P_*algU*_−*lacZ*	Complete P_*algU*_ promoter (541 bp upstream of ATG) EcoRI/HindIII in miniCTX-*lacZ*	(Damron et al. 2009)
pEX100T-NotI	*Pseudomonas* suicide vector with NotI restriction site fuse into SmaI of pEX100T, sacB, oriT, CbR	(Damron et al. 2009)
pEX100T-Δ*algW*	1.4-kb fragment flanking the *algW* (PA4446) gene ligated into pEX100T-NotI with in-frame deletion of *algW*	(Qiu et al. 2007)
pEX100T-Δ*algU*	2.5-kb fragment flanking the *algU* (PA0762) gene ligated into pEX100T-NotI with in-frame deletion of *algU* with 24 bp remaining.	(Damron et al. 2009)
pCR4-*pilA*::Gm^R^	1941 bp fragment contained 966 bp upstream of ATG and 975 bp downstream of TAA with a MluI Gm^R^ cassette (750 bp) inserted 9 bp before ATG of an in-frame deleted *pilA* ligated into pCR4-TOPO	This study
pLP170	8.3-kb, promoterless-*lacZ*, Ap^R^, multiple cloning site	(Preston et al. 1997)
pLP170-P_*algU*_	Complete P_*algU*_ promoter (541 bp upstream of ATG) fused with *lacZ* in pLP170 BamHI/HindIII	This study
pLP170-P_*algD*_	Complete P_*algD*_ promoter (989 bp upstream of ATG) fused with *lacZ* in pLP170 BamHI/HindIII	This study
pLP170-P_*pilA*_	Complete P_*pilA*_ promoter (500 bp upstream of ATG) fused with *lacZ* in pLP170 BamHI/HindIII	This study
pLP170-P_*algW*_	Complete P_*algW*_ promoter (1000 bp upstream of ATG) fused with *lacZ* in pLP170 BamHI/HindIII	This study

* P. Phibbs, East Carolina University, Greenville, NC.

** J. Govan, University of Edinburgh, Scotland, U.K.

### Construction of mutant strains

In-frame deletion of target genes *algU* (PA0762) and *algW* (PA4446) in PAO579 was carried out through polymerase chain reaction (PCR) amplification of the upstream and downstream regions (500–1000 base pairs) flanking the target gene. Using crossover PCR, these upstream and downstream regions were fused and ligated into pEX100T-NotI. A two-step allelic exchange procedure was used by first screening the possible single cross-over mutants for carbenecillin resistance and sucrose sensitivity, then screening for sucrose resistance and carbenecillin sensitivity. For construction of PAO579*rpoN*::Tc^R^ strain, *rpoN* (PA4462) was amplified through PCR, cloned into pCR®4-TOPO® Vector (Invitrogen, Carlsbad, CA) and transformed into *E. coli* DH5α. In vitro transposon mutagenesis was performed on the pCR®4-TOPO®-*rpoN* vector using the EZ::TN <KAN-2> insertion kit (Epicentre Biotechnologies, Madison, WI). The mutant library was recovered and triparentally conjugated into en masse to PAO579. Mutants were selected on PIA with tetracycline and screened for the nonmucoid phenotype. PAO579*pilA::aacC1* strain was constructed using crossover PCR of 1000 bp upstream and downstream fragments of *pilA* (PA4525) containing an internal MluI restriction site. This crossover PCR product was cloned into the pCR®4-TOPO® vector and restriction digested using MluI. A cassette containing a gentamycin resistance marker restriction digested with MluI and ligated into the pCR®4-TOPO®-*pilA* construct. Finally, the pCR®4-TOPO®-*pilA* construct was triparentally conjugated and a two-step allelic exchange procedure was used by first screening for gentamycin resistance and carbenicillin resistance, then gentamycin resistance and carbenicillin sensitivity. All strains were amplified by PCR and sequenced to confirm proper insertion or deletion of target genes.

### Plasmid construction and complementation

Plasmids used in this study are indicated in Table 1. Standard recombinant DNA cloning techniques were used in the construction of all plasmids used in this study (Sambrook and Russell 2001). Briefly, oligonucleotide primers were designed based on PAO1 sequence information and synthesized by Eurofin MWG Operon. Primer sequence information is available upon request. PCR amplifications were done using EasyStart™ Micro 50 PCR Mix-in-a-Tube (Molecular BioProducts, San Diego, CA) and *Taq* DNA Polymerase (New England BioLabs, Ipswich, MA). The pCR®4-TOPO® Vector (Invitrogen) was used as an intermediary before ligation into the target vector. All plasmids were purified using QIAprep® Spin Miniprep Kit (Qiagen Sciences, Hilden, Germany). All plasmid constructs were sequenced to confirm no mutations. Plasmids were transformed into *E. coli* DH5α for all intermediate cloning steps. Completed plasmids were triparentally conjugated into target *P. aeruginosa* strains using pRK2013 as a helper strain (Figurski and Helinski 1979).

### Alginate assay

Alginate was measured using the previously published carbazole reaction (Knutson and Jeanes 1968). Bacterial strains were streaked in triplicate on PIA, and incubated at 37°C for 24 h. The bacterial cells were scraped into 10 mL of phosphate buffered saline (PBS) and the OD_600_ was recorded. The amount of uronic acid was measured and compared with an alginate standard curve made with d-mannuronic acid lactone (Sigma-Aldrich, St. Louis, MO) in the range 0–100 μg/mL. The reported values represent an average of three independent experiments with standard deviation.

### β-galactosidase activity assay

*Pseudomonas* strains carrying the plasmid pLP170 (empty vector) or pLP170 containing P_*algD*_, P_*algU*_, P_*algW*_, or P_*pilA*_ or PAO1 miniCTX-P_*algU*_−*lacZ* and miniCTX-P_*algD*_−*lacZ* with pHERD20T, pHERD20T-*pilA*, or pHERD20T-*pilA*^108^ were cultured at 37°C on three PIA plates supplemented with carbenecillin or carbenicillin and tetracycline. Bacterial cells were harvested, resuspended in PBS and the OD_600_ was recorded. The cells were permeabilized using toluene, and β-galactosidase activity was measured with results calculated and reported in Miller Units (Miller 1972). One Miller Unit equivalent to 1000 ×(A_420_/−1.75 × A_550_/OD_600_/mL/min). The reported values represent an average of three independent experiments with standard deviation. Student's *t*-test was performed to determine statistical significance.

### Protein analyses

Bacterial strains were grown at 37°C on PIA or LB media supplemented with the appropriate antibiotics. Cells were harvest and whole cell lysates were prepared using the ProteaPrep Cell Lysis Kit (Protea Biosciences, Morgantown, WV) and the total protein content was quantified using D_c_ Assay (Bio-Rad, Hercules, CA). Using a hemagglutinin (HA) immunoprecipitation kit ((Thermo Fisher Scientific, Rockford, IL), HA-tagged proteins were isolated by combining cell lysates with anti-HA agarose beads, incubating overnight at 4°C, washing with a tris-buffered saline-Tween solution, and eluting. About, 25 μg of protein samples were boiled for 10 min in Tricine Sample Buffer (Bio-Rad) and electrophoresed on a 16.5% Tris-Tricine gel (Bio-Rad). Samples were then electro-blotted onto a Hybond™-P polyvinylidene difluoride transfer membrane (GE Healthcare, Wauwatosa, WI). The membrane was blocked using 3% skim milk/PBS. Mouse monoclonal antibody for the alpha subunit of RNA polymerase (Neoclone, Madison, WI) and rat monoclonal antibody for HA (Roche Diagnostics, Indianapolis, IN) were used as primary antibodies. Anti-type IVa pilin rabbit polyclonal antibodies were gifted from the Lory laboratory (Harvard Medical School, Cambridge, MA). Horseradish peroxidase-labeled goat anti-mouse IgG, goat anti-rabbit or goat anti-rat IgG were used as secondary antibodies. Primary and secondary antibodies were diluted in 3% skim milk/PBS to 1:5000 and 1:10,000, respectively. Western blot results were imaged using enhanced chemiluminescence Advance Western Blotting Detection Kit (Amersham; GE Healthcare) and UVP BioImagining Systems (Upland, CA). When necessary, blots were stripped using 62.5 mmol/L Tris-HCl pH 6.8, 2% SDS, 100 mmol/L β-mercaptoethanol for 10 min at 40°C.

## Results

### PAO579 has polymorphisms in *algW* and *pilA*

Using strain PAO1 as a reference genome, we performed next-generation sequencing to determine the mutation(s) (*muc-23*) responsible for mucoidy in PAO579 (ALOF00000000) (Withers et al. 2012). As a result, 16 nonsynonymous and 15 synonymous single nucleotide polymorphisms (SNPs) were identified using two criteria: more than 4 × coverage and greater than 60% frequency Table S1). Consistent with previous phenotypic observations (Stanisich and Holloway 1969; Govan and Fyfe 1978), we detected mutations at loci *rpsL* (PA4268) and *leuA* (PA3792), both corresponding to previous genetic and phenotypic markers in the parent strain PAO381 (Table 2). Furthermore, PCR sequencing revealed that two genes, *algW* (PA4446) and *pilA* (PA4525) contained SNPs in PAO579 when compared with strain PAO381. Our results showed a substitution of an adenine for a thymine at nucleotide 715 of the coding region of *algW* (PA4446), ultimately resulting in the exchange of phenylalanine for isoleucine at amino acid 239 (I239F) in AlgW (Table 2). We identify this mutation as *algW*^I239F^. We also observed three tandem nucleotide substitutions (C → T^325^, A → G^326^, G → A^327^) in *pilA* (PA4525) creating a premature stop codon (TGA) (Table 2). The *pilA* gene encodes for the protein precursor that constitutes the type IVa pilin. Furthermore, analysis of these tandem mutations at nucleotides 352–327 revealed a truncation in PilA from 149 to 108 amino acids (Fig. S1). We identify this mutation as *pilA*^108^. We hypothesized that one, or both of these mutations could be responsible for mucoidy in PAO579.

**Table 2:** Summary of sequencing results

SNP	Genome position	Nucleotide change	SNP position (gene size)	Locus tag	Gene	Gene product	Protein change	Domain
1	4771865	T → C	263 (372)	PA4268	*rpsL*	30S ribosomal protein S12	K88R+	16S Binding
2	4251149	G → A	322 (1779)	PA3792	*leuA*	2-isopropylmalate synthase	E108K+	DRE_TIM_LeuA
3	4980548	A → T	715 (1170)	PA4446	*algW*	DegS-like MucA Protease	I239F	Trypsin-L2 Loop
	5069207	C → T	325 (450)			Type IVa pilin precursor		
4	5069206	A → G	326 (450)	PA4525	*pilA*	Type IVa pilin precursor	***	C-terminal FTF
	5069204	G → A	327 (450)			Type IVa pilin precursor		

SNP, synonymous single nucleotide polymorphisms; FTF, phenylalanine-threonine-phenylalanine.

*** Stop Codon.

### *algW* and *algU* are required for alginate overproduction in strain PAO579

**Figure 1:**
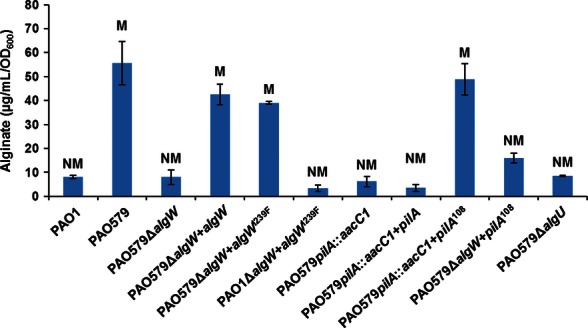
Alginate production by *Pseudomonas aeruginosa* strains PAO1, PAO579, and PAO579 mutants. All strains were grown on *Pseudomonas* Isolation Agar (PIA) plates for 24 h at 37°C then for 24 h at room temperature. The alginate was collected and measured using the carbazole assay. The values are reported as mean ± standard deviation of three independent experiments. M, Mucoid; NM, Nonmucoid.

AlgW is the first in a cascade of proteases responsible for the degradation of MucA (Wood et al. 2006; Cezairliyan and Sauer 2009; Wood and Ohman 2009). To determine if *algW* is required for mucoidy in PAO579, we deleted *algW* and observed a decrease in alginate production and a conversion to the nonmucoid phenotype (Fig. 1). Next, we cloned *algW* and *algW*^I239F^ into the shuttle vector pHERD20T containing the arabinose-inducible P_*BAD*_ promoter (Qiu et al. 2008a). We observed that the expression of *algW* in trans restored mucoidy to PAO579Δ*algW* (Fig. 1). Similarly, we observed that expression *algW*^I239^ in trans could restore mucoidy in PAO579, however we did not observe a significant difference in the amount of alginate produced (Fig. 1). More importantly, *algW*^I239F^ did not induce mucoidy in PAO1Δ*algW* (Fig. 1). These data indicate that AlgW is required for mucoidy in PAO579, however the I239F mutation is not responsible for inducing mucoidy in PAO579.

Previous reports suggested that *algU* was not required for alginate overproduction of strain PAO579 (Boucher et al. 2000). However, our data showing that AlgW is required for mucoidy suggests that MucA degradation, and subsequently, the release of AlgU, is occurring in PAO579. If so, expression of MucA would result in a loss of mucoidy. To test this, *mucA* was expressed from using both pHERD20T and the low-copy number P_*tac*_ vector (Graupner and Wackernagel 2000), and loss of mucoidy was observed (data not shown). Additionally, we observed that deletion of *algU* from PAO579 resulted in a loss of mucoidy (Fig. 1). Expression of *algU* in trans in PAO579Δ*algU* restored mucoidy (data not shown). These data suggest that AlgU is required for alginate production in PAO579.

### Expression of *pilA*^108^ induces mucoidy in PAO579

Since our data suggested that *algW*^I239F^ is not responsible for the induction of mucoidy, we next examined the role of *pilA*^108^ in the regulation of alginate overproduction in PAO579. Based on our sequence analysis, we determined that *pilA*^108^ encodes for ∼11 kDa protein. Western blot analysis using anti-PilA polyclonal antibodies revealed a lack of the full length pilin protein in PAO579 (Fig. S2A). Additionally, HA-tagged PilA108 was only detected with Western blot analysis after immunopurification (Figs. S2B and C). Furthermore, analysis of our sequence data revealed that the C-terminal of PilA^108^ consists of a three amino acid motif of phenylalanine-threonine-phenylalanine (FTF) (Fig. S1). Previously, we reported that the C-terminal motif WVF found on the small envelope protein MucE can induce mucoidy through the activation of AlgW (Qiu et al. 2007). Based on this information, we hypothesized that the truncated *pilA*^108^ could induce mucoidy through AlgW. We tested this hypothesis by first inactivating *pilA* in PAO579 through the insertion of a gentamycin cassette (PAO579*pilA*::*aacC1*). We observed a decrease in alginate production and a conversion to the nonmucoid phenotype in PAO579*pilA*::*aacC1* (Fig. 1). Next, we complemented these experiments by cloning the wild-type *pilA* and *pilA*^108^ into pHERD20T containing the arabinose-inducible P_*BAD*_ promoter and expressed them in trans. We observed that expression of *pilA*^108^ increased alginate production inducing mucoidy in PAO579*pilA*::*aacC1* (Fig. 1), while expression of *pilA* wild-type did not (Fig. 1). Similar results were also observed in PAO1 (data not shown). We also observed that the expression of *pilA*^108^ did not confer mucoidy in PAO579Δ*algW*, suggesting that PilA^108^ acts through AlgW. To confirm whether the FTF-motif found in PilA^108^ can induce mucoidy via AlgW, we cloned the major outer membrane porin precursor *oprF* (PA1777) and *oprF* with the addition of the FTF motif to its C-terminal (*oprF*-FTF) into pHERD20T. Next, we conjugated this construct, as well as pHERD20T-*pilA* and pHERD20T-*pilA*^108^ into PAO1 and PAO1Δ*algW*. After incubating in the presence of 0.1% (w/v) arabinose, we observed *oprF*-FTF and *pilA*^*108*^ increased alginate production and conferred mucoidy in PAO1 (Table 3). Expression of *oprF* did not induce mucoidy in PAO1, which is consistent with our previously published results (Qiu et al. 2008a). Expression of *pilA* did not induce mucoidy in PAO1. As expected, we did not observe any phenotypic change when *pilA*, *pilA*^108^, *oprF*, *oprF*-FTF were expressed in PAO1Δ*algW* (Table 3). These results suggest that the FTF-motif found at the C-terminal of PilA^108^ can activate mucoidy through AlgW.

**Table 3:** Complementation analyses of *pilA*, *pilA*^108^, *oprF*, and *oprF*-FTF

*Pseudomonas* strains	Vector control	*pilA*	*pilA*^*108*^	*oprF*	*oprF-*FTF
PAO1	NM (3.6 ± 0.4)	NM (11.7 ± 1.6)	M (52.7 ± 7.1)	NM (6.3 ± 5.8)	M (40.8 ± 6.8)
PAO1Δ*algW*	NM (6.7 ± 3.2)	NM (4.4 ± 4.4)	NM (5.2 ± 3.3)	NM (4.7 ± 1.9)	NM (7.4 ± 1.2)

NM, nonmucoid; M, mucoid; FTF, phenylalanine-threonine-phenylalanine. pHERD20T was used in this study. All strains were grown on PIA supplemented with 300 μg/mL carbenicillin and 0.1% (w/v) l-arabinose at 37°C for 24 h. The alginate measurements for three independent experiments are represented as (Mean μg of Alginate/mL/OD_600_ ± standard deviation).

### *pilA*^108^ and *algW* are required for proteolytic degradation of MucA

**Figure 2:**
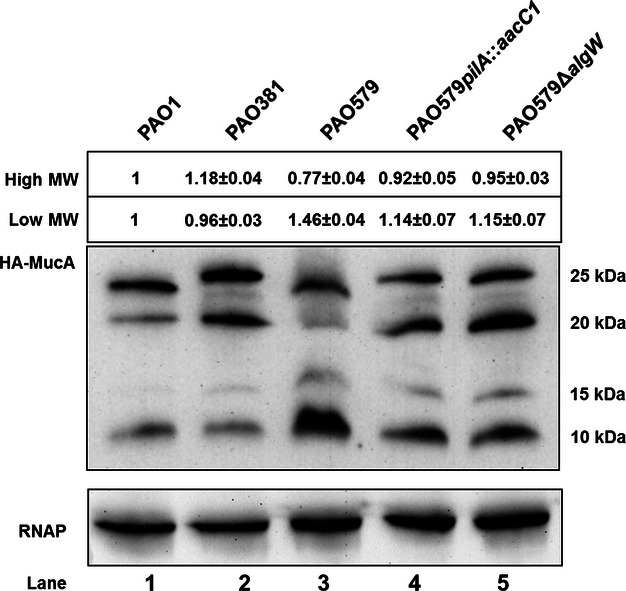
Western blot analysis of N-terminally tagged HA-MucA in PAO1, PAO381, PAO579, PAO579*pilA*::*aacC1*, and PAO579Δ*algW*. Shown are representative panels of three independent experiments. All strains were grown on *Pseudomonas* Isolation Agar (PIA) plates supplemented with carbenicillin and 0.1% arabinose for 24 h at 37°C then for 24 h at room temperature. Cell lysates were prepared and 25 μg of total protein was loaded for each sample for SDS-PAGE electrophoresis. Following transfer, the membrane was immunoblotted with primary rat anti-HA and secondary horseradish peroxidase-labeled goat anti-rat IgG. Protein levels were categorized as High MW (>20 kDa) or Low MW (<20 kDa), normalized to PAO1 pHERD20T-HA-*mucA*, and presented as relative means ± standard deviations.

As expression of *pilA*^108^ required *algW* to confer mucoidy in PAO579, we hypothesized that the activation of alginate production was due to increased MucA degradation. In order to test this hypothesis, we measured the degradation of MucA by expressing an N-terminally HA-tagged MucA (Damron et al. 2009) via the P_*BAD*_ arabinose-inducible promoter (pHERD20T-HA-*mucA*) in PAO1, PAO381, PAO579, PAO579*pilA*::*aacC1* and PAO579Δ*algW*. All strains were cultured on PIA plates supplemented with carbenicillin and 0.1% arabinose. Western blot analysis of PAO1 and PAO381 showed similar levels of full length HA-MucA, although we detected greater accumulation of protein at 20 kDa and 10 kDa in PAO381 (Fig. 2, Lane 1 and 2). We detected a decrease in full length HA-MucA and increase in lower molecular weight products (∼10 kDa) in PAO579 when compared with all other test strains (Fig. 2, Lane 3). We also detected similar amounts of full length HA-MucA in PAO579*pilA*::*aacC1* and PAO579Δ*algW* as PAO381 (Fig. 2, Lane 4 and 5). These results suggest that there is an increase in MucA degradation in PAO579 when compared with its progenitor strains PAO1 and PAO381. Additionally, *pilA*^108^ and *algW* are required for increased MucA degradation in PAO579.

### Increased transcriptional activity at the *P*_*algD*_ and *P*_*algU*_ promoters in PAO579 requires *pilA*^108^, *algW*, *algU*, and *rpoN*

**Figure 3:**
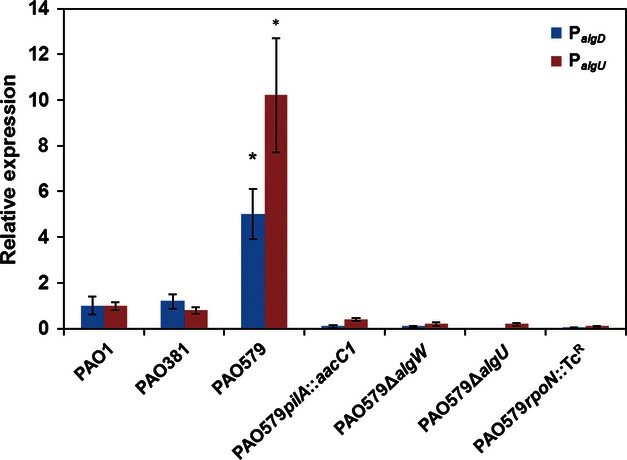
The β-galactosidase activity of the P_*algD*_ and P_*algU*_ promoter fusions was measured using the pLP170-P_*algD*_−*lacZ* and pPLP170-P_*algU*_−*lacZ* reporter constructs. Each strain was on incubated at 37°C on *Pseudomonas* Isolation Agar (PIA) plates supplemented with 300 μg/mL of carbenicillin. The values for the mean ± standard deviation are shown as relative expression, and are representative of three independent experiments. Asterisks indicate statistical significance (*P < 0.05).

Based on our Western blot analyses of MucA, we hypothesized that deletion of *pilA*^108^, *algW*, and *algU* would result in a decrease in transcriptional activity for the alginate biosynthetic operon. To test this, we measured promoter activity by fusing the entire P_*algD*_ promoter to *lacZ* in the plasmid pLP170 (Preston et al. 1997), and performing a Miller assay (Miller 1972). We observed a significant increase in P_*algD*_ activity in PAO579 as compared with its progenitor strains PAO1 and PAO381 (Fig. 3). We also observed a significant decrease in P_*algD*_ activity in the *pilA*^108^, *algW*, and *algU* mutants in PAO579 (Fig. 3). As the expression of AlgU gene is autoregulated, it is possible to indirectly measure the release of AlgU following MucA degradation using a β-galactasidase promoter fusion assay. Similar to our analysis of the *algD* promoter, we used the plasmid pLP170 to fuse the entire *algU* promoter region to *lacZ*, and performed a Miller assay. Similar to our *algD* promoter analysis, we observed a significant increase in P_*algU*_ activity in PAO579 compared to PAO1 and PAO381, and a significant decrease in P_*algU*_ activity in the *pilA*^108^, *algW*, and *algU* mutants (Fig. 3).

**Figure 4:**
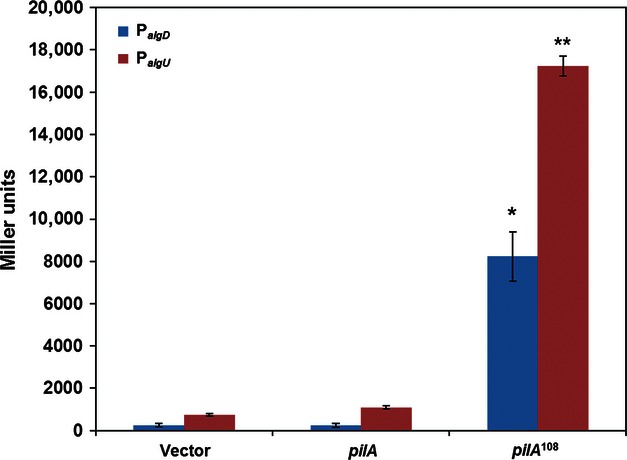
The β-galactosidase activity of the *algD* and *algU* promoters was measured using the miniCTX-P_*algD*_−*lacZ* and miniCTX-P_*algU*_−*lacZ* reporter constructs integrated to *att* site in PAO1 and pHERD20T, pHERD20T-*pilA* or pHERD20T-*pilA*^108^ were conjugated using the helper plasmid pRK2013. Each strain was incubated at 37°C on *Pseudomonas* Isolation Agar (PIA) plates supplemented with tetracycline, carbenicillin, and 0.1% arabinose. The values for the mean and standard deviation are shown as relative expression, and are representative of three independent experiments. Asterisks indicate statistical significance (*P < 0.01; **P < 0.0005).

Additionally, we measured the effect of wild-type *pilA* and *pilA*^108^ expression on merodiploid strains carrying P_*algD*_ and P_*algU*_ fused with the *lacZ* reporter gene (Damron et al. 2009) in the presence of the shuttle vector pHERD20T, pHERD20T-*pilA*, and pHERD20T-*pilA*^108^. After induction with 0.1% arabinose, we observed that expression of *pilA*^108^ caused significant increase in P_*algD*_ activity as compared to the vector control and wild-type *pilA* (Fig. 4). There was no significant difference in P_*algD*_ activity between the vector control and *pilA* wild-type (Fig. 4). A similar trend was observed when measuring the P_*algU*_ promoter activity (Fig. 4).

**Figure 5:**
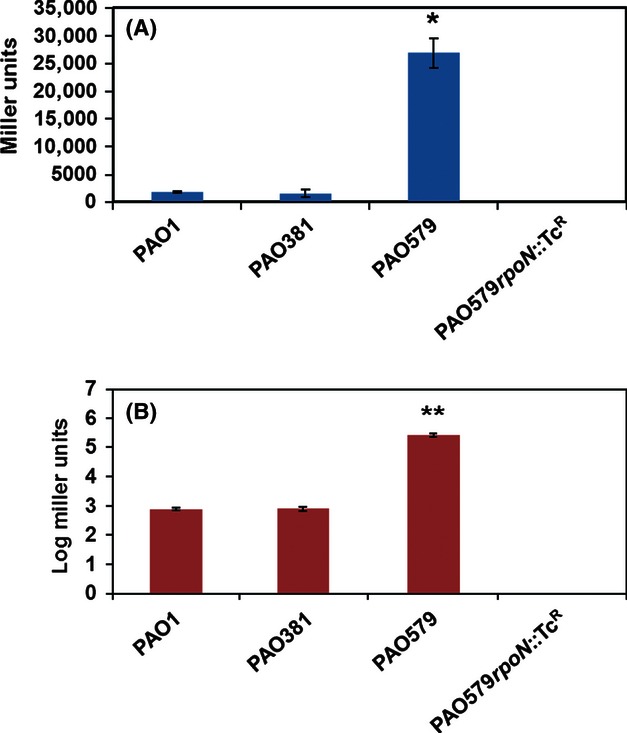
The β-galactosidase activity of the *algW* (A) and *pilA* promoters (B) was measured using the pLP170-P_*algW*_−*lacZ* and pPLP170-P_*pilA*_−*lacZ* reporter constructs. Each strain was incubated at 37°C on *Pseudomonas* Isolation Agar (PIA) plates supplemented with 300 μg/mL of carbenicillin. The values for the mean and standard deviation are representative of three independent experiments. The measurement for P_*pilA*_ activity is presented in log Miller Units. Asterisks indicate statistical significance (*P < 0.005; **P < 0.0005).

Previously, it was reported that the alternative sigma factor RpoN was also required for alginate production in PAO579 (Boucher et al. 2000). Consistent with these findings, we observed that inactivation of *rpoN* in PAO579 (PAO579*rpoN*::Tc^R^) resulted in a significant decreases in activity at the *algD* and *algU* promoters when compared to PAO579 (Fig. 3). Interestingly, overexpression of *rpoN* using pHERD20T failed to induce mucoidy in PAO579*pilA*::*aacC1* and PAO579Δ*algU*, suggesting that RpoN regulates mucoidy in PAO579 upstream of PilA and AlgU. Additionally, we performed Western blot analysis to measure the level of RpoN in PAO1, PAO579, and PAO381. We found the level of RpoN is comparable in these three strains (data not shown). RpoN regulates global gene expression of many motility genes in nonmucoid strains of *P. aeruginosa* (Dasgupta et al. 2003). Likewise, it has been shown that RpoN is responsible for transcription of *pilA* through the PilS/PilR two-component regulatory system (Hobbs et al. 1993). Deletion of *rpoN* from a mucoid strain resulted in dysregulation of ∼20% of the genome (Damron et al. 2012). In this study, it was also shown that RpoN may be involved in expression of *algW* (Damron et al. 2012). Expression of *pilA*^108^ in PAO579 *rpoN*::Tc^R^ did not restore mucoidy indicating *rpoN* may have multiple roles in alginate overproduction in strain PAO579. We hypothesized that the inability of *pilA*^108^ to confer mucoidy in PAO579*rpoN*::Tc^R^ could be due to RpoN role driving transcription at both the *pilA* and *algW* promoters. We tested this hypothesis by measuring the level of promoter activities of P_*pilA*_ and P_*algW*_. The level of P_*pilA*_ and P_*algW*_ activity between strains PAO1 and PAO381 were similar; however, we observed a significant increase in activity in PAO579 at both promoters sites (Figs. 5A and B). The level of promoter activity for both P_*pilA*_ and P_*algW*_ fell below the threshold for detection in PAO579*rpoN*::Tc^R^ (Figs. 5A and B). These results are consistent with previous reports, stating that RpoN drives transcription of *pilA* and *algW* in PAO579. Together, these results suggest that RpoN regulates mucoidy in PAO579 upstream of *pilA*^108^, *algW*, and *algU*.

## Discussion

Generally speaking, there are two types of mucoid isolates found in CF sputum samples: those with mutations mapped within the *mucABCD* cluster (Schurr et al. 1996; Boucher et al. 1997; Anthony et al. 2002), and those with undefined mutations mapped outside of the *mucABCD* cluster. While it is known that *mucA* mutants are associated with chronic infections, it is not clear what mucoid-related genotypes are present in those early colonizing strains. In this study, we used whole genome sequence analysis to identify the unknown positive regulator(s) of alginate production in *P. aeruginosa* strain PAO579 (*muc-23*), an isogeneic derivative of PAO1. We identified three tandem point mutations in the *pilA* gene resulting in a premature stop codon. These alterations cause a truncation in the major subunit of type IVa pilin at amino acid 108. This truncated version of PilA reveals a C-terminal primary amino acid sequence of FTF, which functions as a signal to activate alginate overproduction through the proteolytic degradation of MucA. We observed that the transcriptional activity at the *algD* and *algU* promoters is increased in PAO579, while inactivation of *algW*, *algU*, *rpoN*, and the truncated *pilA* causes a significant decrease in activity at these promoters. Also of note, we determined that the sigma factor RpoN regulates transcription at both the *pilA* and *algW* promoters in PAO579.

Initially we identified a nonsynonymous mutation in *algW* of PAO579 (*algW*^I239F^). However, this mutation did not have an impact on AlgW activity (Fig. 1). Deletion of *algW* in PAO579 did result in a loss of mucoidy, however expression of *algW* and *algW*^I239F^ in trans from the P_*BAD*_ promoter did not result in a significant difference in alginate overproduction (Fig. 1). The amino acid substitution occurs in a nonconserved site next to the L2 loop (Cezairliyan and Sauer 2009) which may explain why we did not observe any significant difference in the amount of alginate produced. Taken together, these results indicate that the activity of AlgW^I239F^ is not increased in comparison to wild type AlgW in activating alginate overproduction. However, the requirement of AlgW for mucoidy does implicate the release of AlgU due to proteolytic degradation of MucA. Western blot analysis of the HA-MucA confirms that there is increase in lower molecular weight products in PAO579 as compared with PAO1, PAO381, PAO579*pilA*::*aacC1*, and PAO579Δ*algW* (Fig. 2). These results indicate that there is increased MucA degradation in PAO579.

**Figure 6:**
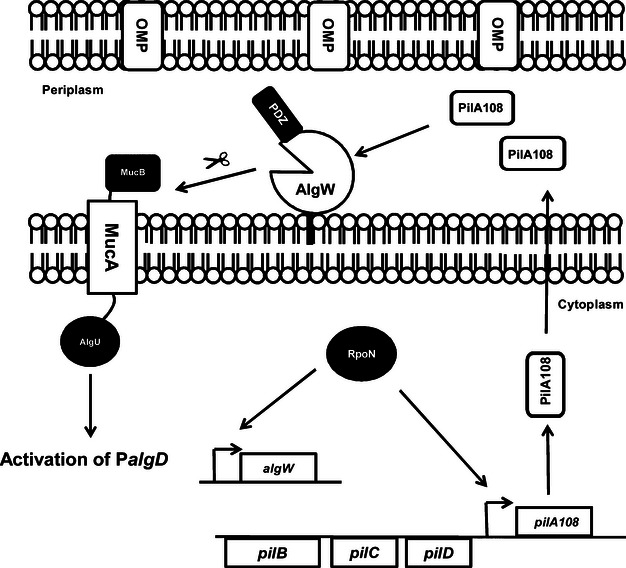
Schematic diagram of summarizing the induction of alginate production and mucoid conversion by PilA in *Pseudomonas aeruginosa* strain PAO579. The sigma factor RpoN is required for transcription of *pilA*^108^ and *algW*. PilA^108^ is transported to the periplasm, where it activates the periplasmic protease AlgW which proteolytically degrades the antisigma factor MucA releasing the sequestered sigma factor AlgU. AlgU drives transcription of the alginate biosynthetic operon via the *algD* promoter.

We observed that the deletion of *algU* resulted in a loss of mucoidy in PAO579 (Fig. 1). In Boucher et al. 2000, RpoN was shown to be involved in driving transcription at P_*algD*_. In this same study, *algU* was inactivated by an insertion of a tetracycline resistance cassette and observed to not be essential for the mucoid phenotype of PAO579. In our study, as AlgW was required for the mucoid phenotype of PAO579, degradation of MucA, and transcriptional activity at the P_*algD*_ promoter (Figs. 1, 2 and 3), we hypothesized that AlgU was most likely required for the mucoid phenotype of PAO579. We then in-frame deleted *algU* from PAO579 and observed this strain to be nonmucoid (Fig. 1). We were also able to complement this strain by expressing *algU* in trans and observed a return to the mucoid phenotype (data not shown). In this respect, the essential difference from our study here and the Boucher et al. study is that *algU* was completely deleted from PAO579 in our study. Although our data argues that *algU* is required, it also confirms that RpoN is required for mucoidy in PAO579. However, overexpression of RpoN in PAO579*pilA*::*aacC1* and PAO579Δ*algU* did not confer mucoidy. Additionally, we observed that *rpoN* may be regulating alginate production upstream of AlgU through controlling expression of *algW* and *pilA* (Fig. 5). All together these data suggest that RpoN act upstream of *pilA*^108^ and algU in regulating mucoidy in PAO579. This pathway is illustrated in Figure 6, RpoN drives transcription of *algW* and *pilA*^108^. PilA^108^ activates AlgW to begin proteolytic degradation of MucA. Upon release, AlgU drive transcription of the alginate biosynthetic and *algUmucABCD* operons via the P_*algD*_ and P_*algU*_, respectively.

The *pilA* gene encodes for the type IV pilin precursor which is responsible adhesion to respiratory epithelial cells (Doig et al. 1988), as well as surface translocation or twitching motility (Mattick 2002). Previously, Yang et al. (2010) showed that two missense mutations in *pilA* of *Myxococcus xanthus* can cause membrane accumulation of pili, resulting in a decrease in exopolysaccharide production. Similarly, the current study shows that three tandem mutations in *pilA* can affect exopolysaccharide production; however we observed an overproduction in alginate (Fig. 1). An increased frequency of mutants has been shown to occur in *P. aeruginosa* strains with mutations in the DNA mismatch repair system such as *mutS* (Oliver et al. 2000). Additionally, alterations in the *mutL* and *uvrD* have also been shown to result in a mutator phenotype (Oliver et al. 2002). However, we did not detect any polymorphisms at these loci, suggesting that the frequency at which three tandem point mutations may occur is quite low. Although the C-termini of pilin displays a high diversity, those found in CF isolates tend to cluster together into one phylogenic group (Kus et al. 2004). Through BLAST searches, we identified 6 clinical isolates that carry an internal FTF motif (Fig. S3). It is known that mucoid mutants are selected for in the CF lung. Our study suggests that mutations can arise in envelope proteins, such as *pilA*, and induce alginate overproduction. Because, regulated proteolysis is controlled by the AlgW protease and envelope proteins, we wonder if a treatment strategy targeting these proteins could block alginate overproduction and allow for better clearance of chronic *P. aeruginosa* infections.

## Supporting Information

**Figure S1.** Diagram showing the predicted structure for PAO1 PilA and PAO579 PilA. (A) Base pair substitutions in the *pilA* gene in PAO579 result in a premature STOP codon and a truncation in the PilA protein from 149 amino acid residues and molecular weight of 15.5 to 108 amino acid protein with a molecular weight of 11.2 kDa. PAO579 PilA has an activating domain (β2) that is responsible for the positive regulation of alginate production which consists of a phenylalanine-threonine-phenylalanine (FTF) motif at its C-terminal. (B) The truncation of PAO579 PilA at amino acid 108 also results in the loss of the β3 and β4 antiparallel sheets, as well as revealing the activating domain in the β2 sheet.

**Figure S2.** Western blot analysis of PilA108 (A) PAO579 and PAO1 were grown at 37°C on PIA. Cellular protein was harvested and was subjected to SDS-PAGE electrophoreses, membrane transfer, and probed with anti-Pilin polyclonal antibodies. (B) PAO1 cells containing pHERD20T (Vector), pHERD20T-*pilA*-HA and pHERD20T-*pilA*108-HA were grown at 37°C on PIA plates supplemented with carbenicillin and 0.1% arabinose. Cellular protein was harvested and was subjected to SDS-PAGE electrophoreses, membrane transfer, and probed with anti-HA monoclonal antibody. (C) PAO1 pHERD20T and pHERD20T-*pilA*108-HA was cultured on PIA plates supplemented with carbenicillin and 0.1% arabinose. Cellular protein was harvested then purified using high affinity anti-HA immunoprecipitation and analyzed using SDS-PAGE electrophoresis, membrane transfer, and probed with anti-HA monoclonal antibody.

**Figure S3.** Basic Local Alignment Search Tool (BLAST) for the C-terminal of the PilA. The red arrow identifies the location of the truncation revealing the phenylalanine-threonine-phenylalanine (FTF) motif found in PAO579. The black arrows identify clinical isolates containing the same internal motif with NCBI accession GI number and strain name.

**Table S1.** Complete summary of sequencing results for PAO579.
