# Supplementary material for: Truncation of type IV pilin induces mucoidy in Pseudomonas aeruginosa strain PAO579
Source: Microbiologyopen. 2013 Mar 27;2(3):459–70. doi: 10.1002/mbo3.86 (PMC3684759; doi:10.1002/mbo3.86)

Supplemental Tables, Figures and Figure Legends

Table S1. Complete summary of sequencing results for PAO579

SNP	Genome Position	Nucleotide Change	SNP Position (Gene Size)	ORF	Gene	Gene Product	Protein Change	Domain	PAO381*
1	5036891	A-C	-183(1602)	PA4500		Probable ABC Transporter			Yes
2	4771865	T-C	263(372)	PA4268	*rpsL*	30S ribosomal protein S12	K88R+	16S binding	Yes
3	183697	T-G	930(939)	PA0159		Probable LysR transcription regulator	C130W		Yes
4	4251149	G-A	322(1779)	PA3792	*leuA*	2-isopropylmalate synthase	E108K+	DRE TIM LeuA	Yes
5	6115455	T-G	858(1254)	PA5434	*mtr*	Tryptophan permease	K286N	Trp permease	Yes
6	4980548	A-T	715(1170)	PA4446	*algW*	DegS-like MucA protease	I239F	Trypsin-L2 Loop	No
7	4924552	C-G	532(837)	PA4394	*yggB*	Conserved hypothetical protein	V178L+	MS Channel	Yes
8	4924553	G-C	531(837)	PA4394	*yggB*	Conserved hypothetical protein			Yes
9	6098781	G-C	1758(3018)	PA5418	*soxA*	Sarcosine oxidase α subunit			ND
10	1871272	T-C	157(342)	PA1728		Hypothetical protein	S53P	DUF 1260	Yes
11	4212201	A-G	1907(2429)	PA3760	*nagF*	Phosphotransferase transporter	H636R	Pyruvate kinase	Yes
12	1589438	G-C	101(1107)	PA1459	*cheB*	Probable methyltransferase	G34A	Signal receiving	Yes
13	6079222	A-G	1179(1962)	PA5399	*dgcB*	Dimethylglycine catabolism			ND
14	5743462	G-C	1292(1680)	PA5100	*hutU*	Urocanase	T431S+	hutU	Yes
15	5743461	C-G	1293(1680)	PA5100	*hutU*	Urocanase			Yes
16	4869855	T-G	474(771)	PA4341		Probable transcription regulator	E158D+		Yes
17	4344266	A-G	570(1296)	PA3877	*nark1*	Nitrite extrusion protein 1			ND

18	721611	C-T	(1200)+55	PA0668	*tyrZ*	Tyrosyl-tRNA synthetase 2			ND
19	2239547	T-G	-280(408)	PA2046		Hypothetical protein			
20	413850	T-C	-196(291)	PA0369		Hypothetical protein			ND
21	4334140	G-C	207(990)	PA3870	*moaA1*	Molybdopterin bios. Protein A1			ND
22	4448855	C-G	(1500)+38	PA3970	*amn*	AMP nucleosidase			ND
23	4448856	G-C	(1500)+37	PA3970	*amn*	AMP nucleosidase			ND
24	1603577	T-G	-94(759)	PA1477	*ccmC*	Heme exporter protein			Yes
25	5069207	G-A	325(450)	PA4525	*pilA*	Type-4 pilin precursor	STOP	DITFTF	No
26	5069206	T-C	326(450)	PA4525	*pilA*	Type-4 pilin precursor	STOP	DITFTF	No
27	5069205	C-T	327(450)	PA4525	*pilA*	Type-4 pilin precursor	STOP	DITFTF	No
28	1440625	A-G	1920(1971)	PA1327		Probable protease			ND
29	169284	G-C	-77(546)	PA0149		Probable σ70 factor			ND
30	2768847	C-G	9958(16884)	PA2462		Hypothetical protein	A3320P	A/DA autotrp shdA	ND
31	2813321	A-G	128(1419)	PA2495	*oprN*	OMP OprN precursor	D43G	TolC	ND

*-present in PAO381; Yes, present in both PAO381 and PAO579; No, only present in PAO579; ND, No data available

Figure S1. Diagram showing the predicted structure for PAO1 PilA and PAO579 PilA. A) Base pair substitutions in the *pilA* gene in PAO579 result in a premature STOP codon and a truncation in the PilA protein from 149 amino acid residues and molecular weight of 15.5 to 108 amino acid protein with a molecular weight of 11.2 kDa. PAO579 PilA has an activating domain (β2) that is responsible for the positive regulation of alginate production which consists of a phenylalanine-threonine-phenylalanine (FTF) motif at its C-terminal. B) The truncation of PAO579 PilA at amino acid 108 also results in the loss of the β3 and β4 antiparallel sheets, as well as revealing the activating domain in the β2 sheet.

A

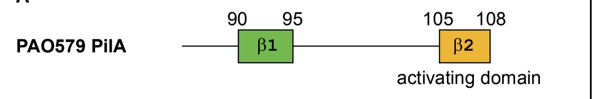

	# amino acids	mw-dalton	pI
PAO1 PilA	149	15,512	6.51
PAO579 PilA	108	11,159	4.72

PAO1 Seq GCG GGT GAT ATT ACC TTT ACC TTC CAG

PAO579 Seq GCG GGT GAT ATT ACC TTT ACC TTC **TGA**

A G D I T F T F *

105 106 107 108

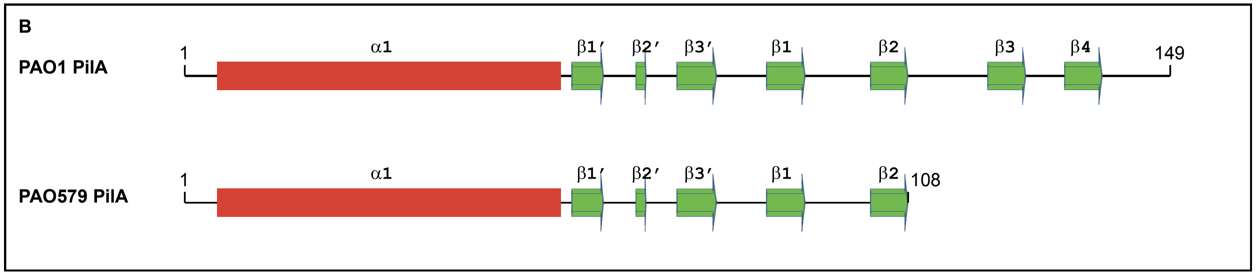

Figure S2. Western blot analysis of PilA108 Panel A) PAO579 and PAO1 were grown at 37°C on PIA. Cellular protein was harvested and was subjected to SDS-PAGE electrophoreses, membrane transfer, and probed with anti-Pilin polyclonal antibody. Panel B) PAO1 cells containing pHERD20T (Vector), pHERD20T-*pilA*-HA and pHERD20T-*pilA*^{108}-HA were grown at 37°C on PIA plates supplemented with carbenicillin and 0.1% arabinose. Cellular protein was harvested was subjected to SDS-PAGE electrophoreses, membrane transfer, and probed with anti-HA monoclonal antibody. Panel C) PAO1 pHERD20T and pHERD20T-*pilA*^{108}-HA was cultured on PIA plates supplemented with carbenicillin and 0.1% arabinose. Cellular protein was harvested then purified using high affinity anti-HA immunoprecipitation and analyzed using SDS-PAGE electrophoresis, membrane transfer, and probed with anti-HA monoclonal antibody.

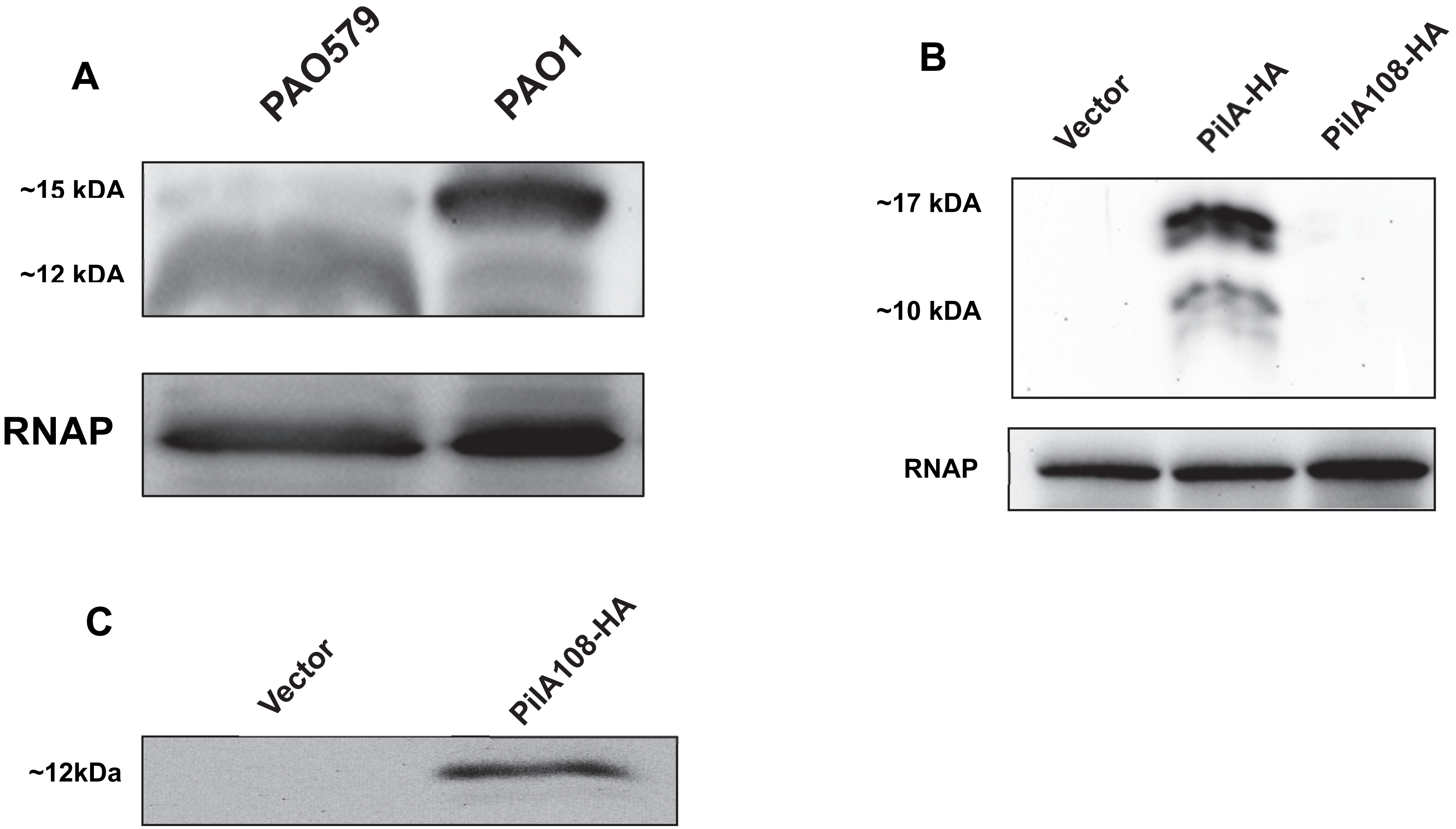

Figure S3. Basic Local Alignment Search Tool (BLAST) for the C-terminal of the PilA. The red arrow identifies the location of the truncation revealing the phenylalanine-threonine-phenylalanine (FTF) motif found in PAO579. The black arrows identify clinical isolates containing the same internal motif with NCBI accession GI number and strain name.

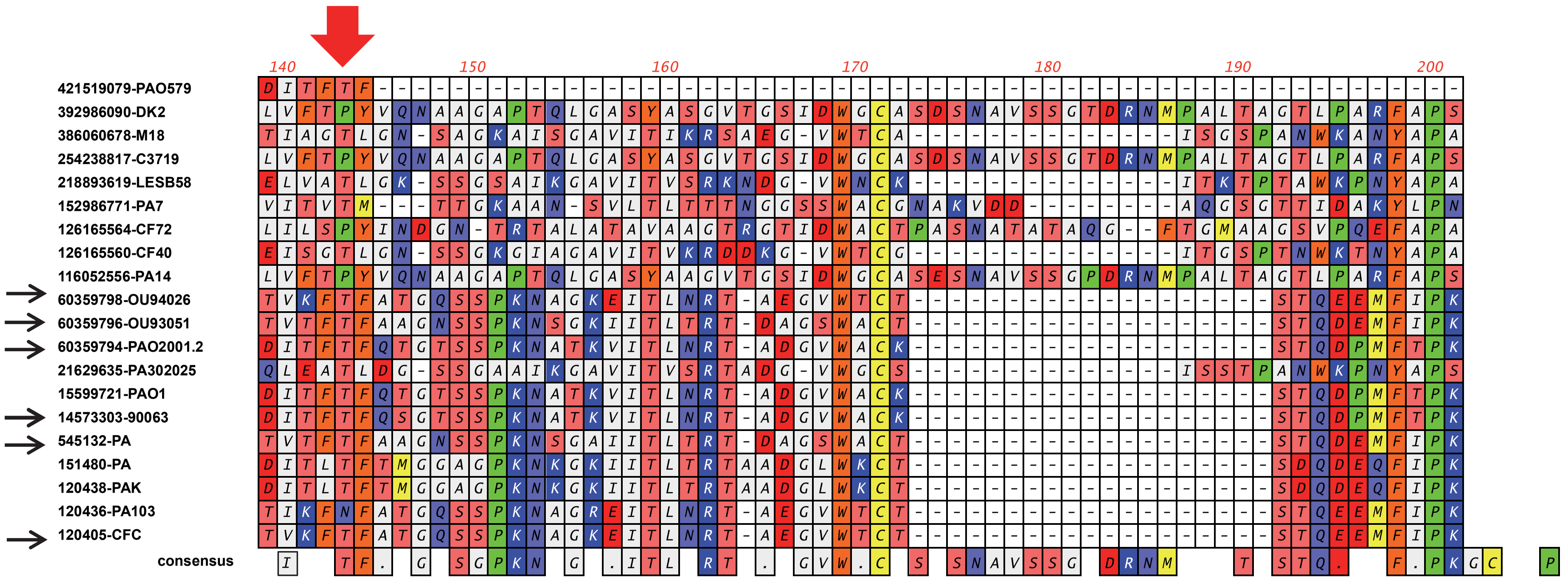

Supplement: Supplementary file 1 [file mbo30002-0459-SD1.pdf]
